# The Protective Role of Astaxanthin for UV-Induced Skin Deterioration in Healthy People—A Randomized, Double-Blind, Placebo-Controlled Trial

**DOI:** 10.3390/nu10070817

**Published:** 2018-06-25

**Authors:** Naoki Ito, Shinobu Seki, Fumitaka Ueda

**Affiliations:** Pharmaceutical and Healthcare Research Laboratories, Research and Development Management Headquarters, FUJIFILM Corporation, 577, Ushijima, Kaisei-machi, Ashigarakami-gun, Kanagawa 258-8577, Japan; shinobu.seki@fujifilm.com (S.S.); fumitaka.ueda@fujifilm.com (F.U.)

**Keywords:** astaxanthin, antioxidant, skin, ultra-violet, UV, MED, moisture

## Abstract

Skin is a major safeguard tissue in humans. Because biological barrier function is deteriorated by several kinds of stresses including exposure to ultra-violet (UV) rays, the protection and treatment of skin conditions by dietary supplements are important. We therefore evaluated the effects of dietary supplementation with an algal food-derived antioxidant, astaxanthin, on UV-induced skin deterioration. Twenty-three healthy Japanese participants were recruited to a 10-week double-blind placebo-controlled study. They were assigned to the astaxanthin group supplemented with a capsule containing 4 mg of astaxanthin or the placebo group. To assess the protective role of astaxanthin for UV-induced skin deterioration, we determined the minimal erythema dose (MED) and analyzed UV-induced changes of moisture and transepidermal water loss (TEWL) at baseline and after 9 weeks of supplementation. Subjective skin conditions were assessed by the visual analog scale. The astaxanthin group showed increased MED compared with placebo. In addition, the astaxanthin group had a reduced loss of skin moisture in the irradiated area compared with placebo. Subjective skin conditions for “improvement of rough skin” and “texture” in non-irradiated areas were significantly improved by astaxanthin. Astaxanthin seems protective against UV-induced skin deterioration and helps maintain healthy skin in healthy people.

## 1. Introduction

The skin, a major safeguard tissue in humans, is composed of the epidermis, dermis and subcutaneous tissue. These structures prevent the invasion of various microorganisms or pathogens and protect our body from physiological damage. The skin also prevents the excessive transpiration of moisture [1]. These protective functions are called “barrier functions”. Impaired barrier functions result in increased transepidermal water loss (TEWL), the loss of water that passes from inside the body through the epidermis, and a decrease of moisture, a water content of the stratum corneum. Furthermore, the skin undergoes oxidative damage by various stresses including daily exposure to ultra-violet (UV) rays from the sun, which leads to the generation of reactive oxygen species (ROS) such as singlet oxygen or secondary lipid peroxyl radicals. These radicals damage biological molecules including proteins or DNA, which disturbs healthy skin conditions. Because the biological barrier function is impaired by these repeated stresses [2] and endogenous anti-oxidative capacity is gradually decreased with age [3], the protection and treatment of skin conditions by daily supplements have received increasing attention [2,4,5,6].

Astaxanthin is a red carotenoid found in shrimp, crab, salmon and microalgae [7,8]. Astaxanthin exerts a strong anti-oxidative activity by scavenging free radicals [9]. In particular, the scavenging effect of astaxanthin for singlet oxygen is approximately 1000 times higher than that of coenzyme Q10 [9]. Astaxanthin is absorbed from the small intestine, transported to the plasma and erythrocytes [10], to the brain by crossing the blood-brain barrier [11,12], and to the skin including the epidermis and dermis [13]. On the basis of its strong anti-oxidative activity, the beneficial effects of astaxanthin as a supplement have been evaluated for a wide variety of human health issues including metabolism [14,15], exercise performance [16], cognitive functions and mental fatigue (combined with or without sesamin) [17,18,19], sleep efficiency (combined with zinc) [20], and skin conditions [21,22,23,24,25]. Because of its strong single oxygen scavenging effects, the protective role of astaxanthin for skin conditions or UV-induced skin deterioration has been evaluated in cells, rodents and humans [13,23,26,27,28,29,30]. Regarding UV-induced skin deterioration, the administration of astaxanthin prevented UV-induced photo-aging and burn-wound progression in rodents [13,27]. In addition to astaxanthin, several carotenoids such as β-carotene and lycopene also showed protective effects against sun damage [2]. However, in contrast to rodent studies, there have been only limited study on the beneficial effects of astaxanthin on UV-induced skin deterioration in healthy people [26].

Sunburn and subsequent darkening is a common response after exposure to sunlight. The beneficial effects of astaxanthin to improve UV-induced skin deterioration in healthy people was previously evaluated [26]. Although supplementation with astaxanthin reduced UV-induced skin darkening as evaluated by colorimetric L value or scoring on a skin tone color scale [26], the change in minimal erythema dose (MED), which is the amount of UV radiation that produces minimal erythema of an individual’s skin [31], and the associated UV-induced changes in skin condition including skin moisture and TEWL have not been analyzed. Thus, although the effects of astaxanthin at relatively late stages of UV-induced skin deterioration including skin color or recovery from UV-induced damage have been analyzed, its effects on the relatively early changes of UV-induced skin deterioration including MED and associated skin conditions are unknown. It is important to determine the effects of astaxanthin at early stages to develop methods to protect skin from UV and maintain healthy skin.

Therefore, in this study, we evaluated the effects of dietary supplementation with astaxanthin on UV-induced skin deterioration. We hypothesized that astaxanthin exerts anti-oxidative activity in the epidermis or dermis to protect skin from UV-induced stimuli. We set MED as the primary outcome and other skin conditions including moisture and TEWL in the irradiated area as secondary outcomes. In addition, subjective skin conditions were analyzed by a visual analog scale (VAS). Safety evaluation was also conducted.

## 2. Materials and Methods

### 2.1. Study Design, Randomization and Blinding

We performed a randomized, double-blind, placebo-controlled, parallel-group comparison trial to evaluate the effects of dietary supplementation with astaxanthin on UV-induced skin deterioration in healthy Japanese participants. This study consisted of 1 week of basement measurement and 9 weeks of supplementation. An equal number of participants was allocated to either the astaxanthin group or placebo group. This study was approved by the Kenshokai Ethical Review Board (Approved Number: 20170927-2) and followed the Declaration of Helsinki and Ethical Guidelines for Medical and Health Research Involving Human Subjects. This study was registered in the UMIN Clinical Trials Registry (ID: UMIN000028925). Participants, practitioners and clinicians were blinded. Practitioners performed interventions, outcome measurement and analysis and clinicians performed safety evaluations. According to our independent trials that evaluated the effects of dietary supplementation with astaxanthin on skin TEWL in 10 healthy people [23], we set the required sample size as 10. We set the evaluation of MED as the primary outcome. We set other skin conditions including moisture and TEWL in the irradiated area and safety evaluation as secondary outcomes. Participants were enrolled and randomly allocated by practitioners into the astaxanthin or placebo group using a random number table considering the sex, age, MED, and moisture and TEWL at non-irradiated areas. Allocation was concealed until all participants finished the tests.

### 2.2. Participants

Participants aged from 30 to less than 60 years in the Osaka area were enrolled in this study. Participants who received an explanation of the objectives and details of this study, and gave written informed consent were included. This study consisted of a supplementation period for 9 weeks from October to December 2017. Participants with the following criteria were included: (1) Subjects aged from 30 to 59 years old at the time informed consent was provided; (2) Subjects whose skin phototype was type II or type III [32,33,34]; (3) Subjects who accepted test for UV-induced erythema in their back skin; (4) Subjects whose basement MED was judged as second, third or fourth points in six-grade UV-irradiated area; (5) Subjects who could visit to the administrative facility on every inspection day; (6) Subjects who provided the written informed consent for the involvement of this trial by themselves. Participants with the following criteria were excluded from the study: (1) Subjects having photosensitivity disorder; (2) Subjects who took medicine which affect light sensitivity of skin; (3) Subjects who regularly went to a dermatology office; (4) Subjects who continuously took a functional food or a quasi-medicine which had same or similar effects with astaxanthin; (5) Subjects who continuously took medicine, quasi-medicine, functional food or supplement which advocated or emphasized effectiveness for which was evaluated in this trial, or which advocated or emphasized the improvement of joint pain; (6) Subjects who had skin disease or abnormality in skin condition such as atopic dermatitis; (7) Subjects who showed the apparent change of skin condition which was not related to the intake of test food at the end of trial compared with the initiation; (8) Subjects who took anti-inflammatory medicine at least once a month; (9) Subjects who worked on the night shift or the day and night shift; (10) Subjects who were receiving the medical treatment or the prophylactic treatment, or who are diagnosed the need of medical treatment; (11) Subjects who had a past history for the severe disease or abnormality of glucose metabolism, lipid metabolism, liver function, kidney function, cardiovascular system including heart function, respiratory tract, endocrine system and nerve system, or for psychiatric disorder; (12) Subjects who had a past history of alcoholism or drug addiction; (13) Subjects who had a risk for food allergy; (14) Subjects who frequently ingested food which was rich in same active ingredient of test food, or who ingested these kind of food during 3 days before and after trial initiation and the last 3 days from the end of trial; (15) Subjects who frequently ingested food which might affect skin color; (16) Subjects who showed apparent abnormality in blood test, or who were positive for HBs antigen or HCV antibody in trial duration including the screening period; (17) Subjects who were pregnant or during lactation when the informed consent was provided, or who hoped to become pregnant during the trial; (18) Subjects who were involved in another trial within 4 weeks prior to this trial, or who will participate in another trial; (19) Subjects who were judged to be inappropriate for this trial by the doctor who was responsible for this trial.

### 2.3. Supplement Formulation

One supplementary capsule contained 4 mg of astaxanthin. The placebo capsule contained a filling agent instead of astaxanthin. One capsule was administered every day for 9 weeks. The astaxanthin capsule and placebo capsule were not distinguishable by their shape, taste or color. For the astaxanthin capsule, we used natural astaxanthin derived from *Haematococcus pluvialis* (ASTOTS, FUJIFILM), which was processed by the dispersant technology that improved the absorbability of astaxanthin in humans [35]. Furthermore, astaxanthin was extracted by supercritical CO2 extraction technology, which enabled us to use solvent-free products.

### 2.4. Evaluation of UV-Induced Skin Deterioration

To assess the effect of UV on skin, we used the back skin for the area to be evaluated because it usually receives less exposure to daily sunlight. A Solar Simulator (Model 601-300 2.5 UV Multiport, Solar Light Co. Inc., Glenside, PA, USA) was used for irradiation. To measure MED, 31.8, 36.5, 42.0, 48.3, 55.5 and 63.9 mJ/cm^2^ of UV-B were used before and after 9 weeks of supplementation. The evaluation of MED was performed at 16–24 h after irradiation. The day after irradiation, an expert evaluator measured the MED by visual assessment. Skin moisture and TEWL at the irradiated area were measured by a Corneometer^®^ (Courage & Khazaka Electronic GmbH, Köln, Germany) and VAPOSCAN AS-VT100RS (Asch Japan Co., Ltd., Tokyo, Japan), respectively. The back skin exposed to 1.15 MED (which meant 1.15 times the amount of UV rays of MED) and 1.32 MED were evaluated for moisture and TEWL. These values were measured at 1 and 7 days after irradiation. A non-irradiated area near the irradiated area was used for normalization. Skin conditions were evaluated in an environment testing room with a stable temperature (21 ± 1 °C) and humidity (50 ± 5%). These objective skin conditions were evaluated before and after supplementation. For weekly subjective skin conditions, the VAS analysis for “skin texture”, “skin clarity”, “youthfulness, visual impression”, “improvement of rough skin”, “improvement of crow’s feet”, “improvement of skin dullness” and “improvement of nasolabial folds” was used.

### 2.5. Blood Sampling and Safety Evaluation

Serum was obtained from the participants at baseline and after 9 weeks of supplementation to perform general biochemical examination of blood including aspartate aminotransferase, alanine transaminase, γ-glutamyl transpeptidase, alkaline phosphatase, lactate dehydrogenase, total bilirubin, direct bilirubin, indirect bilirubin, total protein, albumin, urea nitrogen, creatinine, uric acid, total cholesterol, low-density lipoprotein cholesterol, high-density lipoprotein cholesterol, triglyceride, glucose, sodium, chloride, potassium, HBs antigen and HCV antigen, and hematologic tests including white blood cell, red blood cell, hemoglobin, hematocrit and platelet for safety evaluation.

### 2.6. Statistical Analysis

All results were presented as the mean ± standard deviation (SD). The differences in MED and other scores between placebo and the astaxanthin group were assessed by the Mann–Whitney *U*-test and unpaired *t*-test, respectively. No additional analyses were performed. Probabilities less than 5% (*, *p* < 0.05, **, *p* < 0.01 and ***, *p* < 0.001) were considered statistically significant. Statistical analyses were performed with IBM SPSS statistics software (version 23, IBM Japan, Ltd., Tokyo, Japan).

## 3. Results

### 3.1. Participants

**Figure 1:**
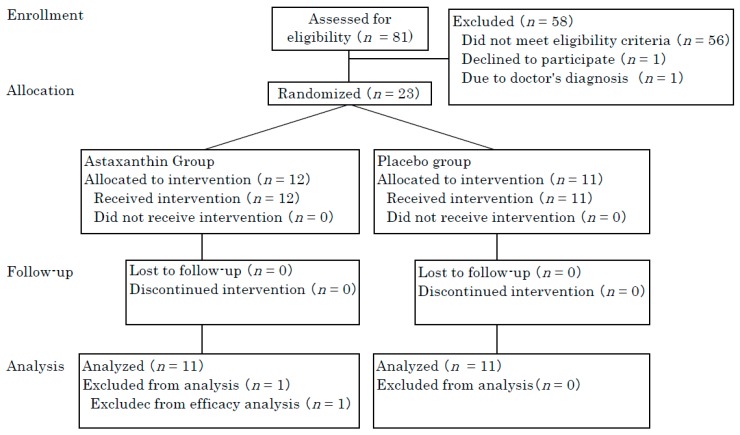
Flow diagram of participants.

Eighty-one participants were recruited from the Osaka area and twenty-three participants (age ranging from 30 to 56 years, 21 females and 2 males) were enrolled. These participants were assigned to the astaxanthin group (*n* = 12) or placebo group (*n* = 11). All participants finished the study. One participant in the astaxanthin group was excluded from the analysis because of an aberrant serum bilirubin level before and after supplementation (Figure 1). Finally, 22 participants (age range from 30 to 56 years, 20 females and 2 males) were analyzed. Thus, the per protocol set analysis was performed. No statistically significant differences were observed for baseline scores including age, gender, MED and moisture and TEWL at the non-irradiated area between the originally included participants and finally analyzed participants. The participants were recruited from September to October 2017. This study consisted of a 9-week administration period from October to December 2017. The placebo group and astaxanthin group were matched according to age, gender, MED, and moisture and TEWL at the non-irradiated area (Table 1). The mean compliance was 100 ± 0% and 99.5 ± 1.7% in the placebo and astaxanthin groups, respectively. All subjects had a >94% ingestion rate. No statistically significant differences were observed for ingestion rate between the groups.

### 3.2. UV-Induced Changes of Skin Conditions

**Figure 2:**
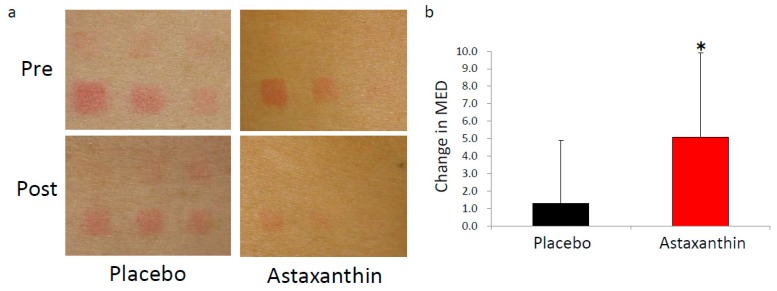
Dietary supplementation with astaxanthin increases the minimal erythema dose. (**a**) Representative imaging of an irradiated area in the placebo group (left) and astaxanthin group (right) before and after supplementation; (**b**) Change in MED from baseline in the placebo group (black) and astaxanthin group (red). * *p* < 0.05 by Mann–Whitney *U*-test. Error bars indicate the standard deviation (SD).

**Figure 3:**
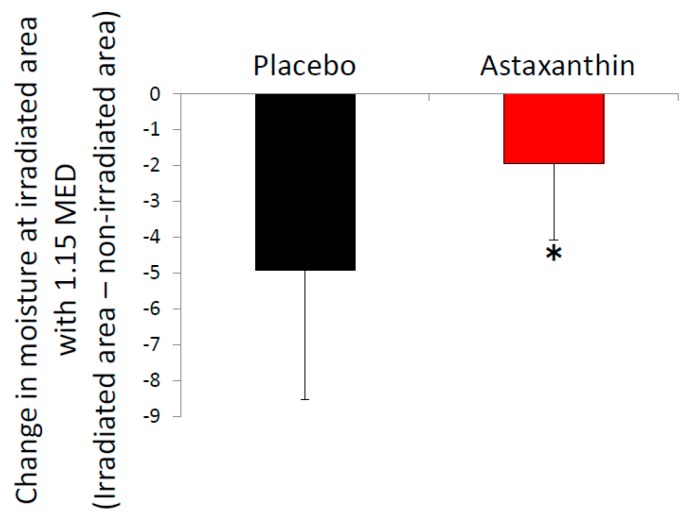
Dietary supplementation with astaxanthin attenuates the decrease of moisture at the irradiated area. Change in moisture from baseline at the irradiated area 7 days after irradiation. The moisture levels at the irradiated area were normalized by those at the non-irradiated area. * *p* < 0.05 by unpaired *t*-test. Error bars indicate the SD.

**Figure 4:**
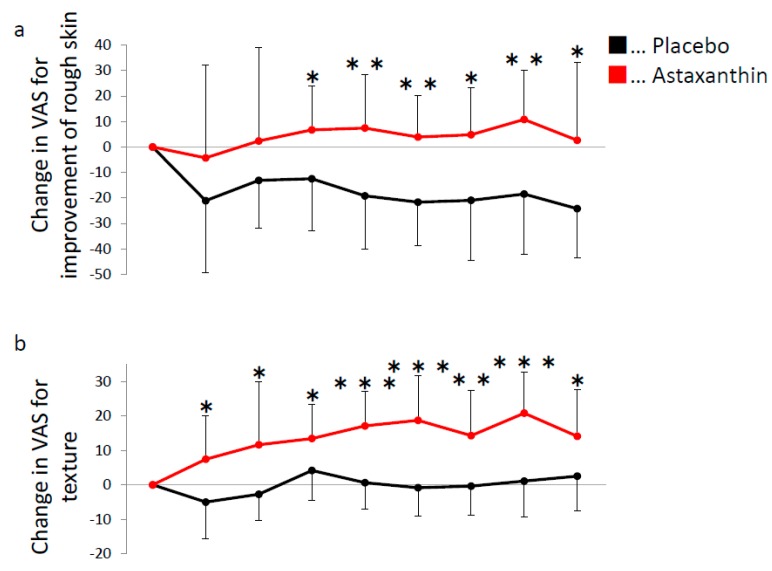
Subjective skin conditions “improvement of rough skin” and “texture” are improved by dietary supplementation with astaxanthin. Change in VAS score for “improvement of rough skin” (**a**) and “texture” (**b**) from baseline at the non-irradiated area in the placebo group (black) and astaxanthin group (red). Positive and negative numbers indicate improvement and deterioration, respectively. * *p* < 0.05, ** *p* < 0.01 and *** *p* < 0.001 by unpaired *t*-test. Error bars indicate the SD.

The aim of this study was to evaluate the effects of dietary supplementation with astaxanthin on UV-induced skin deterioration. We irradiated skin with UV before and after 9 weeks of supplementation. Astaxanthin was administered for 9 weeks starting from the end of the baseline measurements. The raw value of MED was comparable between the astaxanthin group and placebo group before (Table 1) and after supplementation (44.0 ± 6.2 mJ/cm^2^ in the placebo group and 48.4 ± 8.2 mJ/cm^2^ in the astaxanthin group, *p* = 0.162). However, the astaxanthin group showed a significant increase in MED from baseline compared with the placebo group after supplementation (Figure 2a,b). Furthermore, the decrease of moisture at the area irradiated with 1.15 MED was significantly attenuated in the astaxanthin group compared with the placebo group at 7 days after irradiation (Figure 3). The comparable decrease of moisture was observed in the area irradiated with 1.32 MED between the placebo group and astaxanthin group. No significant differences between the astaxanthin group and the placebo group in TEWL at the irradiated area were observed. In addition to objective skin conditions, we also evaluated subjective skin conditions by VAS. Changes from baseline in “improvement of rough skin” and “texture” in the non-irradiated area during the supplementation period were significantly improved in the astaxanthin group compared with the placebo group (Figure 4). There were no significant differences in “skin clarity”, “youthfulness, visual impression”, “improvement of crow’s feet”, “improvement of skin dullness” and “improvement of nasolabial folds”.

### 3.3. Clinical Safety

We observed no adverse events or severe changes in the scores of general biochemical examinations of blood and hematologic tests. Adverse events related to the ingestion of astaxanthin were not observed. Thus, the responsible physician reported no problems with the safety of astaxanthin.

## 4. Discussion

To the best of our knowledge, this is the first report to show the effects of dietary supplementation with astaxanthin on MED and the maintenance of moisture in an irradiated area in healthy subjects. The beneficial role of astaxanthin for skin has been analyzed in several human studies [22,23]. Especially for UV-induced skin deterioration, the protective role of astaxanthin against UV ray have been reported both in vitro [4,7,8] and in vivo [13,27]. Furthermore, the effects of dietary supplementation with astaxanthin on UV-induced skin color change were previously reported [26]. Although a relatively late stage of UV-induced change of skin condition including darkening was analyzed, the effects of dietary supplementation with astaxanthin on the early stage of UV-induced skin change including MED was not reported. We found that dietary supplementation with astaxanthin increased the MED and attenuated the UV-induced decrease of moisture in healthy human. These results demonstrated the protective role of dietary supplementation with astaxanthin against UV-induced stimuli and its usefulness for the maintenance of healthy skin. We observed a mean MED increase of approximately 5 mJ/cm^2^ in the astaxanthin group (Figure 2b). According to the Japan Meteorological Agency, the mean UV-B radiation dose in the Tsukuba area in July between 1994 and 2008 was 23.56 kJ/m^2^/month [36]. Thus, dietary supplementation with astaxanthin might protect skin from damage caused by UV rays comparable to exposure to the sun for 1.5 h in the Japanese summer.

Singlet oxygen is a major oxidant produced by UV rays. Because absorbed astaxanthin reaches the epidermis and dermis [13], and has strong singlet oxygen scavenging activity [9], it is assumed that astaxanthin directly exerts anti-oxidative activity at the epidermis and dermis to protect skin from UV-induced skin deterioration. In addition to MED, we observed an attenuation of the UV-induced decrease of moisture by supplementation with astaxanthin, which was consistent with a previous study reporting an improvement in the UV-induced decrease of natural moisturizing factors in hairless mice by astaxanthin [13]. The administration of astaxanthin also prevented the in vivo UV-induced production of lipid peroxide and upregulation of ROS-producing enzymes, xanthine oxidase and NADPH oxidase 4 [27]. Furthermore, the administration of astaxanthin prevented the UV-induced decrease in the expression of endogenous antioxidant enzymes such as superoxide dismutase and glutathione peroxidase [27]. This suggested that astaxanthin promotes endogenous anti-oxidative effects to reduce the UV-induced activation of ROS-producing enzymes. In addition to its anti-oxidative capacity, astaxanthin has anti-inflammatory effects. Indeed, treatment with astaxanthin prevented the UV-induced increase of interleukin (IL)-1α, IL-6, IL-8 and tumor necrosis factor (TNF)-α in vitro [28] and myeloperoxidase, TNF-α, IL-1β and IL-6 in vivo [27]. In addition, treatment with astaxanthin prevented UV-induced DNA damage and apoptosis in vitro [30,37]. Furthermore, an earlier study showed the beneficial effects of astaxanthin for the treatment of atopic dermatitis [38]. Our study is in agreement with this report for the improvement of VAS regarding “improvement of rough skin” and “texture”. These pleiotropic protective effects might contribute to the prevention of UV-induced skin deterioration observed in this study. Furthermore, as described in the Materials and Methods, we used dispersant technology, which improved the absorbability of astaxanthin in humans indicating this technology reinforced the effects of astaxanthin on UV-induced skin deterioration [35].

Our study had several limitations. Astaxanthin is present in shrimp, salmon and salmon roe, which are preferred by Japanese people. In this study, we prohibited participants to take functional foods or quasi-medicines that contained astaxanthin or other factors with similar effects with astaxanthin. However, we did not estimate the exact dietary intake of astaxanthin by participants. Thus, the effects of supplementary astaxanthin may have been obscured. We hypothesized that astaxanthin supplementation had antioxidant effects. Indeed, the MED was increased by supplementation with astaxanthin. However, the precise mechanisms involved in how astaxanthin improved UV-induced skin deterioration in healthy humans was not elucidated. Furthermore, the relationship between plasma and/or skin astaxanthin concentration and the protective effects against UV-induced skin deterioration was not analyzed. Further studies are required to determine the protective functions of astaxanthin in human skin.

## Figures and Tables

**Table 1:** Baseline characteristics of participants who completed the 10 weeks study.

	Placebo (*n* = 11)	Astaxanthin (*n* = 11)	*p* Value
Age (mean ± SD)	43.7 ± 7.4	43.2 ± 6.6	0.86
Female, *n* (%)	10 (90.1)	10 (90.1)	
MED (mJ/cm^2^) (mean ± SD)	42.7 ± 3.98	43.3 ± 4.27	0.75
Moisture (A.U.) (mean ± SD)	42.5 ± 9.44	43.1 ± 4.95	0.85
TEWL (g/m^2^) (mean ± SD)	3.62 ± 1.42	3.44 ± 0.66	0.72
